# Regulatory frameworks and evidence requirements for traditional, complementary and integrative medicines 

**DOI:** 10.2471/BLT.25.293437

**Published:** 2025-09-03

**Authors:** Qi Chen, Hao-Jin Cheng, Zhao-Xiang Bian, Ai-Ping Lyu, Yue Yang, Kam Wa Chan

**Affiliations:** a School of Pharmaceutical Sciences, Tsinghua University, Beijing, China.; b School of Chinese Medicine, Hong Kong Baptist University, 7 Baptist University Road, Kowloon Tong, Hong Kong SAR, China.; c Vincent V.C. Woo Chinese Medicine Clinical Research Institute, School of Chinese Medicine, Hong Kong SAR, China.

## Abstract

Traditional, complementary and integrative medicine plays an important role in global health-care systems. Despite its widespread use and recognition by more than 170 Member States of the World Health Organization, many disparities in regulation exist between countries. We conducted a comparative analysis of the regulatory frameworks governing traditional medicine products in six high- or middle-income countries or jurisdictions where traditional medicine is used extensively: Australia, Canada, China, Republic of Korea, United States of America and the European Union. We focused on marketing authorization pathways, approval standards and successful approvals. We found differences in regulatory approaches, with countries adopting either clinical study-based or traditional knowledge-based pathways which led to varying requirements for non-clinical and clinical evidence. While the European Union and the United States acknowledge historical human-use evidence, relatively rigorous clinical investigations are required. Australia and Canada consider historical human-use evidence in marketing authorization for products that do not require professional supervision. Recent regulatory reforms in countries such as China and the Republic of Korea aim to enhance regulatory supervision. Across all jurisdictions, fluctuations in the number of successful applications persisted amid evolving policy changes and regulatory requirements. To promote the worldwide use of traditional medicine products, a globally coordinated, tiered and risk-based international framework is needed to ensure the efficacy, quality and safety of traditional medicine products. This approach requires establishing stable (i.e. predictable and consistently implemented) regulatory systems, strengthening the evidence on traditional medicine products with both clinical and real-world data, and facilitating regulatory convergence through reciprocity and globally harmonized evaluation standards.

## Résumé

Les médecines traditionnelles, complémentaires et intégratives jouent un rôle important dans les systèmes de santé mondiaux. Malgré leur utilisation répandue et leur reconnaissance par plus de 170 États membres de l’Organisation mondiale de la Santé, il existe de nombreuses disparités entre les pays en matière de réglementation. Nous avons mené une analyse comparative des cadres réglementaires régissant les produits de médecine traditionnelle dans six pays ou juridictions à revenu élevé ou intermédiaire qui recourent largement à de la médecine traditionnelle : l’Australie, le Canada, la Chine, la République de Corée, les États-Unis d’Amérique et l’Union européenne. Nous nous sommes concentrés sur les mécanismes d’autorisation de mise sur le marché, les normes d’approbation et les autorisations accordées. Nous avons constaté des différences dans les approches réglementaires, les pays adoptant des mécanismes fondés soit sur des études cliniques, sur les connaissances traditionnelles, ce qui a conduit à des exigences variables en matière de preuves cliniques et non cliniques. Si l’Union européenne et les États-Unis reconnaissent les preuves historiques d’utilisation chez l’être humain, des investigations cliniques relativement rigoureuses sont toutefois requises. Pour leur part, l’Australie et le Canada prennent en compte les preuves historiques d’utilisation chez l’être humain dans l’autorisation de mise sur le marché des produits qui ne nécessitent pas de supervision professionnelle. Les récentes réformes réglementaires dans des pays tels que la Chine et la République de Corée visent à renforcer la supervision réglementaire. Dans toutes les juridictions, les fluctuations du nombre de demandes acceptées ont persisté dans un contexte d’évolution des politiques et des exigences réglementaires. Afin de promouvoir l’utilisation de produits de médecine traditionnelle à l’échelle mondiale, un cadre international coordonné à l’échelle mondiale, à plusieurs niveaux et basé sur les risques est nécessaire pour garantir l’efficacité, la qualité et la sécurité de ces produits. Cette approche nécessite la mise en place de systèmes réglementaires stables (c’est-à-dire prévisibles et appliqués de manière cohérente), le renforcement des preuves relatives aux produits de médecine traditionnelle à l’aide de données cliniques et réelles, ainsi que la facilitation de la convergence réglementaire grâce à la réciprocité et à des normes d’évaluation harmonisées à l’échelle mondiale.

## Resumen

Las medicinas tradicionales, complementarias e integrativas desempeñan una función importante en los sistemas de salud a nivel mundial. A pesar de su uso generalizado y del reconocimiento por más de 170 Estados Miembros de la Organización Mundial de la Salud, existen numerosas disparidades en la regulación entre países. Se realizó un análisis comparativo de los marcos regulatorios que rigen los productos de medicina tradicional en seis países o jurisdicciones de ingresos altos o medios donde la medicina tradicional se utiliza extensamente: Australia, Canadá, China, República de Corea, Estados Unidos de América y la Unión Europea. El análisis se centró en las vías de autorización de comercialización, las normas de aprobación y las autorizaciones concedidas con éxito. Se encontraron diferencias en los enfoques regulatorios, con países que adoptaron vías basadas en estudios clínicos o en conocimientos tradicionales, lo que dio lugar a distintos requisitos de evidencia no clínica y clínica. Mientras que la Unión Europea y los Estados Unidos reconocen la evidencia de uso histórico en humanos, se requieren investigaciones clínicas relativamente rigurosas. Australia y Canadá consideran la evidencia de uso histórico en humanos en la autorización de comercialización de productos que no requieren supervisión profesional. Las reformas regulatorias recientes en países como China y la República de Corea tienen como objetivo reforzar la supervisión regulatoria. En todas las jurisdicciones, se observaron fluctuaciones en el número de solicitudes aprobadas con éxito, en un contexto de cambios de política y requisitos regulatorios en evolución. Para promover el uso mundial de productos de medicina tradicional, se necesita un marco internacional coordinado, escalonado y basado en el riesgo que garantice la eficacia, la calidad y la seguridad de los productos de medicina tradicional. Este enfoque requiere establecer sistemas regulatorios estables (es decir, predecibles y aplicados de manera coherente), fortalecer la evidencia sobre los productos de medicina tradicional con datos tanto clínicos como del mundo real y facilitar la convergencia regulatoria mediante la reciprocidad y normas de evaluación armonizadas a nivel global.

## ملخص

يلعب الطب التقليدي والتكميلي والتكاملي دورًا هامًا في أنظمة الرعاية الصحية العالمية. وعلى الرغم من استخدامه على نطاق واسع واعتراف أكثر من 170 دولة عضوًا في منظمة الصحة العالمية به، إلا أن هناك تفاوتات كبيرة في التنظيم بين الدول. لقد أجرينا تحليلًا مقارنًا لأطر العمل التنظيمية التي تتحكم في منتجات الطب التقليدي في ست دول ذات دخل عالي أو متوسط، أو ولايات قضائية حيث يُستخدم الطب التقليدي على نطاق واسع: أستراليا، وكندا، والصين، وجمهورية كوريا، والولايات المتحدة الأمريكية، والاتحاد الأوروبي (EU). لقد ركزنا على مسارات ترخيص التسويق، ومعايير الموافقة، والموافقات الناجحة. وجدنا اختلافات في الأساليب التنظيمية، حيث تتبنى الدول إما مسارات قائمة على الدراسات الإكلينيكية، أو مسارات قائمة على المعرفة التقليدية، مما أدى إلى متطلبات متفاوتة للأدلة الإكلينيكية وغير الإكلينيكية. وبينما يُقرّ كل من الاتحاد الأوروبي والولايات المتحدة بالأدلة التاريخية للاستخدام البشري، إلا أنهما يتطلبان تحقيقات إكلينيكية دقيقة نسبيًا. تأخذ كل من أستراليا وكندا بعين الاعتبار الأدلة التاريخية للاستخدام البشري عند ترخيص تسويق المنتجات التي لا تتطلب إشرافًا مهنيًا. وتهدف الإصلاحات التنظيمية الأخيرة في دول مثل الصين وجمهورية كوريا إلى تعزيز الإشراف التنظيمي. وفي جميع الولايات القضائية، استمرت التقلبات في عدد الطلبات الناجحة في ظل التغيرات السياسية والمتطلبات التنظيمية المتطورة. ولتعزيز الاستخدام العالمي لمنتجات الطب التقليدي، يلزم وجود إطار عمل دولي منسق عالميًا، ومتعدد المستويات، وقائم على المخاطر، لضمان فعالية وجودة وسلامة منتجات الطب التقليدي. ويتطلب هذا الأسلوب إرساء أنظمة رقابية مستقرة (أي قابلة للتنبؤ بها ومطبقة باستمرار)، وتعزيز الأدلة المتعلقة بمنتجات الطب التقليدي من خلال البيانات الإكلينيكية والواقعية، وتسهيل التقارب التنظيمي من خلال المعاملة بالمثل، ومعايير تقييم منسقة عالميًا.

## 摘要

传统、补充和整合医学在全球卫生保健系统中发挥着非常重要的作用。尽管这些医学得到了 170 多个世卫组织成员国的广泛使用和认可，但各国在监管方面的差异仍然很大。我们对以下六个广泛运用传统医学的中高收入国家或司法管辖区的传统医学产品相关监管框架进行了比较分析：澳大利亚、大韩民国、加拿大、美利坚合众国、欧盟和中国。我们重点研究了上市许可途径、审批标准和成功获批相关资料。我们发现了在监管方法方面存在的差异，有的国家采用基于临床研究的途径，而有的国家则采用基于传统知识的途径，从而导致非临床和临床证据相关要求各不相同。虽然美国和欧盟认可历史人用证据，但仍然要求提供相对严格的临床研究数据。澳大利亚和加拿大在对无需专业人员监督的产品进行上市许可审批时却会考虑历史人用证据。大韩民国和中国等国家最近开展的监管改革旨在加强监管机构的监督力度。在政策不断变更且监管要求不断变化的情况下，所有司法管辖区成功申请上市的产品数量始终起伏不定。为了推动在全球范围内广泛使用传统医学产品，必须构建基于风险且分层的全球协调国际框架，以确保传统医学产品的疗效、质量和安全性。该方法要求构建稳定的（即可预测并可持续实施的）监管系统，使用临床和真实世界数据有力支持传统医学产品相关证据，以及基于互惠原则采用全球协调的评估标准来推动实现监管趋同。

## Резюме

Традиционная, комплементарная и интегративная медицина играет важную роль в мировых системах здравоохранения. Несмотря на ее широкое применение и признание более чем 170 государствами-членами Всемирной организации здравоохранения, между законодательствами различных стран существуют значительные расхождения в этом вопросе. Авторы провели сравнительный анализ нормативно-правовой базы, регламентирующей применение препаратов традиционной медицины в шести странах или регионах с высоким и средним уровнем дохода, где широко распространена традиционная медицина: Австралии, Европейском союзе (ЕС), Канаде, Китае, Республике Корея и Соединенных Штатах Америки. Основное внимание уделялось способам государственной регистрации таких препаратов, стандартам их одобрения и успешным случаям получения одобрения. Авторы отметили различия в регуляторных подходах, то есть одни страны используют подходы, основанные на клинических исследованиях, а другие – на традиционных знаниях, что и привело к различиям в требованиях в отношении доклинической и клинической доказательной базы. Хотя ЕС и Соединенные Штаты Америки признают исторические данные о применении препаратов у людей, в этих странах требуется проведение весьма строгих клинических исследований. Австралия и Канада учитывают исторический опыт применения у людей при выдаче государственной регистрации для препаратов, которые не требуют профессионального наблюдения. Недавние реформы нормативно-правовой базы в таких странах, как Китай и Республика Корея, имеют целью усиление регуляторного надзора. Во всех регионах сохранялись колебания в количестве успешно полученных одобрений с учетом изменяющихся и постоянно развивающихся правил и регуляторных требований. Для содействия использованию во всем мире препаратов традиционной медицины необходима международная база, координируемая на глобальном уровне, с различными уровнями допуска и с опорой на оценку риска – только таким способом можно гарантировать эффективность, качество и безопасность таких препаратов. Этот подход требует создания стабильной (то есть предсказуемой и единообразно внедряемой) регуляторной системы, укрепления доказательной базы препаратов традиционной медицины за счет как клинических данных, так и данных, собираемых в условиях реального применения, и содействия сближению регуляторных систем за счет принципа взаимности и гармонизированных в мировом масштабе стандартов оценивания.

## Introduction

Traditional, complementary and integrative medicine (hereafter called traditional medicine) encompasses a diverse range of medical theories and practices that are indigenous to local culture or not conventionally integrated worldwide.^1^^,^^2^ These systems play an important role in global health-care systems by managing both noncommunicable and communicable diseases.^3^ Of the 179 World Health Organization (WHO) Member States providing information to the *WHO global report on traditional and complementary medicine 2019*, 170 acknowledge the use of traditional medicines: 98 countries have established national policies on these products, 109 have enacted national laws and 124 have implemented regulations on herbal medicine.^4^ Beyond its cultural significance, traditional medicine is an effective option for some conditions and is gaining attention along with the development of personalized and system medicine.^5^ The WHO strategy on traditional medicine (2014–2023) advocated integration of traditional medicine into conventional health-care systems to address different global health needs,^6^ which has been expanded in the 2024–2035 strategy.^2^

**Fig. 1:**
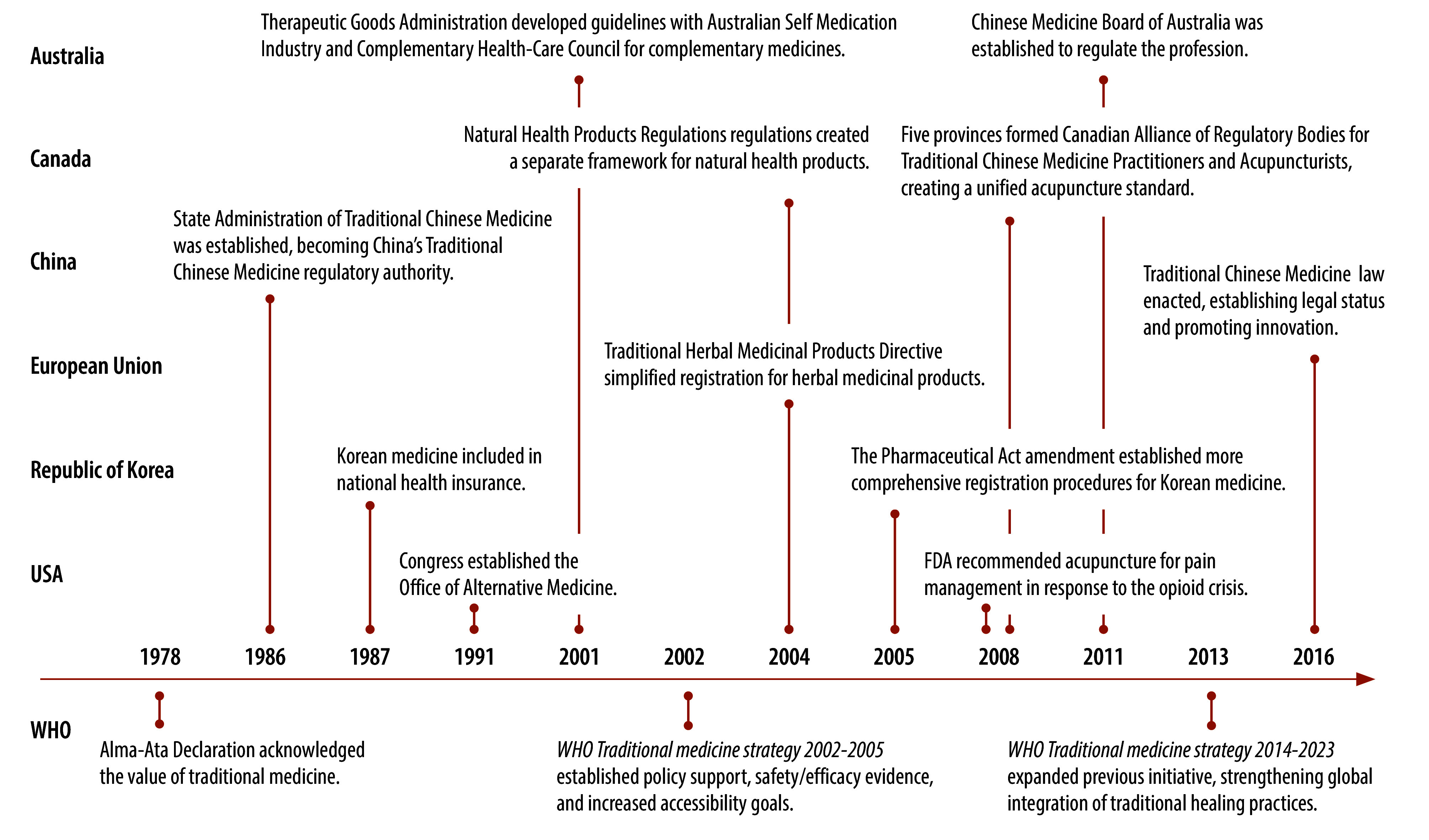
Global regulatory milestones in traditional medicine

FDA: Food and Drug Administration; WHO: World Health Organization.

Many traditional medicine systems trace their use back hundreds or thousands of years. Traditional Chinese medicine was established more than 2000 years ago as documented in the *Huangdi neijing*.^7^^,^^8^ Medicine from the Republic of Korea, which shares roots with traditional Chinese medicine, was codified during the 17th century through texts including the *Dongui bogam*.^9^ Complementary therapies such as homeopathy and naturopathy emerged in Europe after the 18th century. Despite their different origins and epistemology, traditional medicine systems have been increasingly integrated into contemporary health-care frameworks through regulation and scientific evaluation (Fig. 1). Nevertheless, many challenges remain in regulating their market access.^4^

While the European Medicines Agency coordinates certain procedures for marketing authorization across the European Union, traditional medicines are predominately regulated at the national level. Currently, there are no formal reciprocity mechanisms between major jurisdictions for the mutual recognition of registration dossiers (including quality, safety and efficacy data). Only scattered unilateral recognition practices exist. For instance, Health Canada recognizes evidence based on pharmacopoeias from other jurisdictions for the regulation of natural health products. Although WHO has been advocating for evidence-based integration of so-called proven traditional practices into national health systems since the 1970s,^2^ there is no unified standard for incorporating and assessing the evidence from human practices in the evaluation process.^10^ Variations in national policies increase the effort required for multinational registration and mutual recognition.^11^

We analysed the regulatory frameworks, standards for marketing authorization and the number of approvals granted for traditional medicines in five countries (Australia, Canada, China, Republic of Korea and United States of America) and the European Union, where traditional medicine is being extensively used. Through a comparative analysis of global regulatory approaches, we propose strategies to foster international harmonization and collaboration, thereby ensuring the safety, efficacy and patient-centred development of traditional medicine.

## Methods

We focused on traditional medicine products regulated as medicinal products and excluded products classified as food or dietary supplements, except for Canada where natural health products include both categories. We collected data from two sources: (i) official regulatory documents and guidelines of governments and international organizations; and (ii) a literature review. We reviewed the regulatory frameworks and assessed key aspects such as general regulatory approaches, marketing authorization pathways, regulatory requirements for quality control, non-clinical testing and clinical study. The role of historical data on human use in regulatory decision-making was then examined according to the level of evidence.^12^^,^^13^ We categorized regulatory evidence requirements into substantive clinical evidence and supportive evidence. Substantive clinical evidence refers to the data mandated by regulatory authorities to demonstrate the efficacy and safety of a product for its intended use. Supportive evidence refers to data that provide supplementary information for regulatory decision-making but that are not scientifically rigorous enough to independently establish the product’s efficacy and safety. Finally, we extracted and reviewed data on successful marketing authorization applications submitted by sponsors to national regulatory authorities from government official websites, regulatory milestones and regulatory evolution (online repository).^14^

## Results

### Regulatory framework

Regulatory frameworks for traditional medicine products differ considerably across jurisdictions (Table 1). Traditional medicine substances were generally categorized into seven subgroups including animal-based, microbial, mineral-based, nutritional, plant-based, synthetic or processed materials and other substances for regulation (online repository**)**.^14^ Australia’s complementary medicines track and Canada’s natural health products track cover substances from all sources; while the herbal medicinal products track of the European Union, the botanical drugs pathway of the United States of America and the herbal medicinal preparations track of the Republic of Korea focus on plant-based substances.

**Table 1:** Regulatory frameworks for traditional medicines, Australia, Canada, China, Republic of Korea, United States of America and the European Union

Country or jurisdiction	**Product category**	**Scope**	**Regulatory agency**	**Acts and regulations**	**No. of guidance documents^a^**	**General regulatory approaches**	**Marketing authorization pathways**	**Post-market product type**
Australia	Complementary medicines	Herbal, traditional medicines, homeopathic remedies, anthroposophic medicine, essential oils, vitamins and minerals, nutrients, essences (from flowers, shells, gemstones or crystals)	Therapeutic Goods Administration	Therapeutic Goods Regulations 1990	31	No specific pathway; regulated in a similar way to biomedical drugs based on risk assessment	Listed medicines and listed assessed medicines; registered medicines	Non-prescription
Canada	Natural health products	Probiotics, herbal remedies, vitamins and minerals, homeopathic medicines, traditional medicines, other products (e.g. amino acids and essential fatty acids)	Natural and Non-prescription Health Products Directorate	Natural Health Products Regulations	33	Separate marketing pathway; based on monographs of the Natural and Non-prescription Health Products Directorate	Class I (monograph-based); class II or III (traditional and non-traditional)	Non-prescription (self-care products)
China	Traditional Chinese medicines	Plants, animals, minerals, microorganisms, synthetic or processed materials, human-derived products	National Medical Products Administration	Traditional Chinese medicine law and Drug administration law	83	Separate marketing pathway; based on traditional Chinese medicine theory, evidence from human use and clinical study	Traditional pathway (category 3.1); traditional and scientific pathways (category 1.1, 3.2); scientific pathway (category 1.2, 1.3)	Prescription
European Union	Herbal medicinal products	Herbal substances (whole, fragmented or cut plants, plant parts, algae, fungi or lichens), certain exudates	European Medicines Agency and individual agencies of Member States	Herbal Medicinal Products Directive (2001/83/EC)	41	Simplified registration pathway; based on traditional use	Traditional use registration; well-established use marketing authorization; stand-alone or mixed application	Prescription and non-prescription
Republic of Korea	Herbal medicinal preparations	Raw herbal materials, extracts, and traditional formulations specified in recognized herbal medicine books	Ministry of Food and Drug Safety	Pharmaceutical Affairs Act	23	Special application categories; based on traditional literatures and scientific evidence	Drug substance listed in pharmacopoeia of the Republic of Korea; herbal drug requiring data submission; new drugs	Prescription and non-prescription
United States	Botanical drugs	Plant materials, algae, macro fungi and their combinations^b^	Food and Drug Administration	Food, Drug, and Cosmetic Act	1	No specific pathway; regulated as products based on scientific evidence	Over-the-counter drug monographs; new drug application	Prescription and non-prescription

^a^ See online repository for details.^14^

^b^ Excludes preparations containing animals or animal body parts and/or minerals, genetically modified or fermentation-derived single molecular entities and highly purified substances.

Note: data sourced from official regulatory guidelines and government databases.

Each jurisdiction has an independent regulatory approach (regulatory guidelines are given in online repository),^14^ marketing authorization pathways and post-market product type for traditional medicine products. Complementary medicines (three pathways) in Australia and natural health products (three pathways) in Canada are regulated as separate categories in parallel with the conventional prescription and non-prescription drug frameworks, enabling their use without the supervision of a health worker. In China, three pathways are used to regulate traditional Chinese medicines: the traditional pathway (category 3.1) for ancient classical products listed in the official catalogue (currently comprising 317 products); the traditional and scientific pathway (category 1.1, category 3.2) for new formulations or modified ancient classical products combining historical use and scientific research; and the scientific pathway (category 1.2, category 1.3) for novel traditional medicine products requiring comprehensive scientific evidence (online repository).^14^ These products are initially approved only as prescription drugs, with the option to apply for non-prescription status after marketing. Herbal medicinal products (three pathways) in the European Union, herbal preparations (three pathways) in the Republic of Korea and botanical drugs (two pathways) in the United States are integrated into their existing pharmaceutical regulatory frameworks, allowing final products to be marketed as both prescription and non-prescription products (Table 1). The definitions of each marketing authorization pathway across jurisdictions are given in the online repository.^14^

### Quality control regulations

The quality control of traditional medicine products follows the principles applicable to general medicinal products, including good manufacturing practice. All jurisdictions have reference standards regulating the quality of traditional medicines (Table 2). However, due to the complexity of their ingredients and significant batch-to-batch variability, jurisdictions adopt different approaches to the control of raw materials, active constituents, manufacturing, excipients and stability (Table 2). Australia has developed a quantified-by-input approach,^15^ which allows exemption from specific quantitative determination for each batch. Canada adheres to minimum quality-control principles for natural health products.^16^ China adopts comprehensive quality-control requirements, although detailed specifications are unclear. The European Union has a well-established framework for quality control of herbal products.^17^ The Republic of Korea requires that any unquantifiable parameters (e.g. traditional ingredients or process variables that cannot be measured precisely) must be demonstrated to have no effect on product quality, safety and efficacy. The United States imposes the most stringent quality-control requirements for botanical drugs. The country requires rigorous control of raw botanical materials, chemical testing and manufacturing process control, as well as the use of biological assays to ensure consistent therapeutic effects. These requirements form the core of the so-called totality-of-evidence approach of regulation.^18^

**Table 2:** Quality control of traditional medicine products, Australia, Canada, China, Republic of Korea, United States of America and the European Union

**Quality control dimension**	**Australia**	**Canada**	**China**	**European Union**	**Republic of Korea**	**United States**
Main reference standards	Default standards^a^	Natural health products ingredients database; monographs of the Natural and Non-prescription Health Products Directorate; international pharmacopoeias	Chinese Pharmacopoeia	European Pharmacopoeia	Pharmacopoeia and Herbal Pharmacopoeia of the Republic of Korea	United States Pharmacopoeia – National Formulary
Control of crude materials	Requires: Australian approved name; traceability to primary sources; compliance with good agricultural and collection practice	Characterization based on sourcing and harvesting practices; adherence to good agricultural and collection practice	Compliance with good agricultural practice for herbal material production	Detailed botanical identification; geographic origin; compliance with good agricultural and collection practice	Structural identification; compliance with internationally recognized good agricultural and collection practice	Identification through voucher specimens; compliance with good agricultural and collection practice and current good manufacturing practice
Active constituents control	Active constituents may be quantified by input^b^	Batch-to-batch consistency using active or analytical markers	Detailed profiling of key ingredients in compound formulations; fingerprinting and bioactivity studies encouraged	Provision of known therapeutic active constituents, active markers, or analytical markers (without therapeutic activity)	Provision of data on all active constituents; justification required if quantification is not feasible	Multicomponent analysis allowed; biological assays developed for unknown active constituents
Manufacturing process control	Partial batch rotation testing allowed based on quantified by input^b^	Quality control implemented across all stages for complex products	Focus on quality of intermediates, including physicochemical properties, bioactivity and consistency	Detailed quality control tests at intermediate manufacturing stages	Submission of content and analytical methods for all batches, including unknown chemical structures	Quality control tests on every batch
Excipient and contaminant control	Inactive minor excipients must be approved substances; additional good manufacturing practice steps required for proprietary ingredients	Microbial and chemical contaminant limits specified	Systematic analysis of exogenous contaminants; microbial testing based on dosage form; limits for toxic constituents	Compliance with impurity management of the International Council for Harmonization Q3D impurity management; microbial contamination testing based on the European pharmacopoeia	Compliance with the Pharmacopoeia of the Republic of Korea and other official compendia recognized within the Republic of Korea; control of contaminants, residues and packaging integrity	Testing for elemental impurities, residual solvents and radiological contaminants; microbial limit testing
Stability studies	Degradation studies required when full testing is not feasible; selective monitoring of grouped components allowed	Stability studies required to assess the effect of environmental and intrinsic factors (e.g. interactions and degradation) on shelf life	Parameters must reflect internal quality changes; stability determined via long-term and accelerated testing	Stability testing required for active constituents and degradation products; stability-indicating tests (e.g. thin layer chromatography and high-performance liquid chromatography) for unknown active constituents	Long-term and accelerated studies required for stability and shelf life; over-the-counter drugs may be exempt from additional tests under specific conditions, with a maximum shelf life of 36 months	Stability studies required to evaluate degradation products and their toxicity; designed as per International Council for Harmonization Q1A(R2) guidelines to determine shelf life

^a^ Including British Pharmacopoeia, European Pharmacopoeia and United States Pharmacopoeia – National Formulary.

^b^ Quantified by input refers to a manufacturing practice where the content of an ingredient is estimated based on the quantity added during production, rather than assayed in the final product.

Note: data sourced from official regulatory guidelines and government databases.

### Non-clinical and clinical evidence

**Fig. 2:**
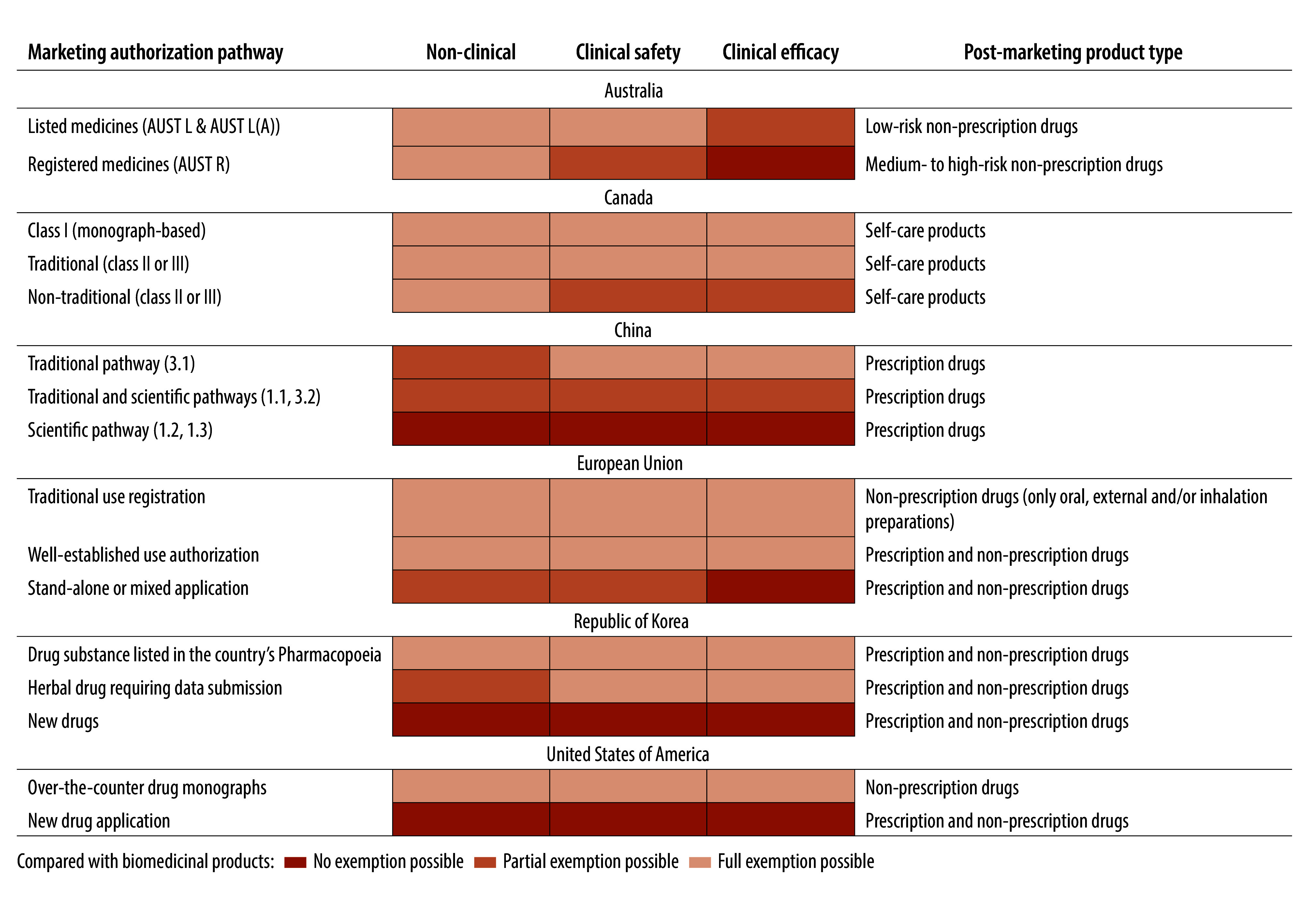
Evidence required to register traditional medicines in different jurisdictions compared with biomedical products

Note: data were retrieved from publicly available official sources (details provided in online repository).^14^

Despite the widespread use of traditional medicine products and the availability of a substantial amount of clinical data, the use of such evidence in the evaluation processes differs considerably across jurisdictions. As a result, exemptions for non-clinical testing, and clinical safety and efficacy studies vary for traditional medicine products compared with biomedical products (Fig. 2; Table 3; available at: https://www.who.int/publications/journals/bulletin/ and online repository).^14^

**Table 3:** Evidence required to register traditional medicines in different jurisdictions

Marketing authorization pathway	Non-clinical	Clinical safety	Clinical efficacy
**Australia**			
Listed medicines (AUST L & AUST L(A))	Not required	Sponsors responsible for determining safety	Efficacy for AUST L determined by sponsors with post-marketing reviews by Therapeutic Goods Administration. AUST L(A) requires premarketing assessment by Therapeutic Goods Administration with traditional and scientific evidence based on indication specificity
Registered medicines (AUST R)	Non-clinical testing may be waived if sufficient human-use evidence exists, supported by comprehensive references aligning with claimed indications, dosage and administration routes	All clinical safety trials, both published and unpublished, are evaluated, with possible exemptions if ingredients meet established quality and safety standards	Clinical efficacy evaluation includes all available data, with traditional use or scientific literature supporting registration, but clinical study data is mandatory
**Canada**			
Class I (monograph-based)	Must meet a single monograph standard of the Natural Health Products Regulations	Not required	Not required
Traditional (class II or III)	Not required	Traditional-use evidence over at least two generations is required, with thorough review of all available data to address safety concerns; additional evidence needed if risks identified	Requires pharmacopoeia evidence or at least two independent references
Non-traditional (class II or III)	Not required	Risk-based assessment determines clinical requirements, ranging from phase II or phase III trials to observational and literature-based studies based on risk level	Risk-based assessment determines clinical requirements, ranging from phase II or phase III trials to observational and literature-based studies based on risk level
**China**			
Traditional pathway (3.1)	Non-clinical efficacy studies are exempt, but safety studies are required, following the Catalogue of Ancient Classical Prescriptions and corresponding standards	Not required	Not required
Traditional and scientific pathways (1.1, 3.2)	Non-clinical efficacy studies may be exempt if human-use experience sufficiently supports key clinical aspects, but non-clinical safety studies remain mandatory	Human experience data must be evaluated for adequacy and applicability, with thorough analysis of adverse events and identification of potential toxic risks based on traditional Chinese medicine theories	Traditional Chinese Medicine product registration relies on a combination of theoretical support, historical human experience and prospective clinical studies, including randomized controlled trials, pragmatic trials and observational studies
Scientific pathway (1.2, 1.3)	Comprehensive non-clinical efficacy and safety studies are mandatory	Full clinical safety trials are required	Clinical efficacy must be demonstrated through at least two well-controlled studies
**European Union**			
Traditional use registration	Simplified registration with 30 years of traditional use, requiring bibliographic reviews and expert reports, with additional tests only if safety concerns arise	Bibliographic and expert sources must confirm long-term human use with no significant safety concerns, and data gaps must be identified	Traditional-use evidence must demonstrate efficacy without indications of harm under normal usage conditions
Well-established use authorization	Requires ≥ 10 years of well-documented use in the European Union with published literature, which allows exemptions from new non-clinical tests if justified	Comprehensive literature reviews covering pre- and post-marketing studies and epidemiological evidence are needed with missing information addressed	Systematic scientific evaluation must demonstrate consistent conclusions on safety, efficacy and applicability, with at least one high-quality clinical study typically required
Stand-alone or mixed application	Requires both literature and new non-clinical tests, with exemptions allowed if adequately justified	Clinical safety trials are required unless literature sufficiently justifies exemptions	At least two well-designed randomized controlled trials are required to demonstrate clinical efficacy
**Republic of Korea**			
Drug substance listed in the country’s Pharmacopoeia	Not required	Not required	Not required
Herbal drug requiring data submission	Non-clinical testing requirements vary depending on prescription type; exemptions apply to new processing methods, while different toxicity studies are required for modifications, literature-based prescriptions, and reported adverse reactions	Clinical safety studies are generally not required, except in cases where serious adverse reactions have been reported	Not required
New drugs	Comprehensive toxicology and pharmacology data required for approval	Full clinical safety trials are mandatory; with exemptions for exploratory studies if safety is supported by other data	Confirmatory efficacy trials are required
**United States**			
Over-the-counter drug monographs	Must comply with the United States Pharmacopeia and National Formulary standards	Safety is established through generally recognized as safe and effective (GRASE) criteria and post-marketing evidence	Efficacy is based on published and unpublished studies
New drug application	Non-clinical data may be reduced for early-phase trials, but full data are required for final approval	Full clinical safety trials are required similar to other drug products	Full clinical efficacy trials are required with at least two well-controlled studies

Note: data were retrieved from publicly available official sources (details provided in online repository).^14^

For non-clinical investigations, regulatory requirements across all jurisdictions generally allow partial or full exemptions for traditional medicine products based on existing evidence from human use and product risk levels. Non-clinical testing can be fully exempted in Australia (complementary medicines track) and Canada (natural health products track) for non-prescription drugs with evidence supporting long-standing human use. In China, newly approved traditional Chinese medicine products can only be registered with a higher-risk prescription-only status. Only limited exemptions are possible for non-clinical efficacy tests. In the European Union, all three marketing authorization pathways recognize human-use evidence for non-clinical testing, but the required evidence levels vary according to the duration of human use and the type of evidence (scientific or traditional theory). The Republic of Korea provides a comprehensive framework that evaluates the credibility of human-use evidence, offering pathways for partial or full exemptions for non-clinical tests. In the United States, non-clinical testing exemptions are permitted under the over-the-counter drug monograph pathway (which is challenging to navigate).^18^ Full non-clinical testing is still mandatory under the new drug application pathway.

For clinical safety and efficacy studies, all jurisdictions recognize historical human-use literature as the source of clinical evidence, but the requirement for additional clinical studies varies. Australia accepts historical literature to support authorization but mandates clinical studies for higher-risk complementary medicines, particularly for those products with specific higher-risk therapeutic indications (e.g. prevention, cure or alleviation of a serious form of a disease, ailment, defect or injury). Canada fully accepts historical human-use literature and exempts additional clinical studies when the evidence is considered sufficient. In the European Union, the stand-alone or mixed application pathway requires rigorous clinical studies, although literature may be used as support when further studies are considered unnecessary or inapplicable. In the United States, marketing authorization under the new drug application pathway requires clinical safety and efficacy studies equivalent to those required for biomedical products, emphasizing dose–response and multiple-batch clinical data to ensure consistency. Both China and the Republic of Korea provide comprehensive pathways that allow partial or complete exemption from clinical studies based on historical human-use evidence.

### Levels of clinical evidence 

**Fig. 3:**
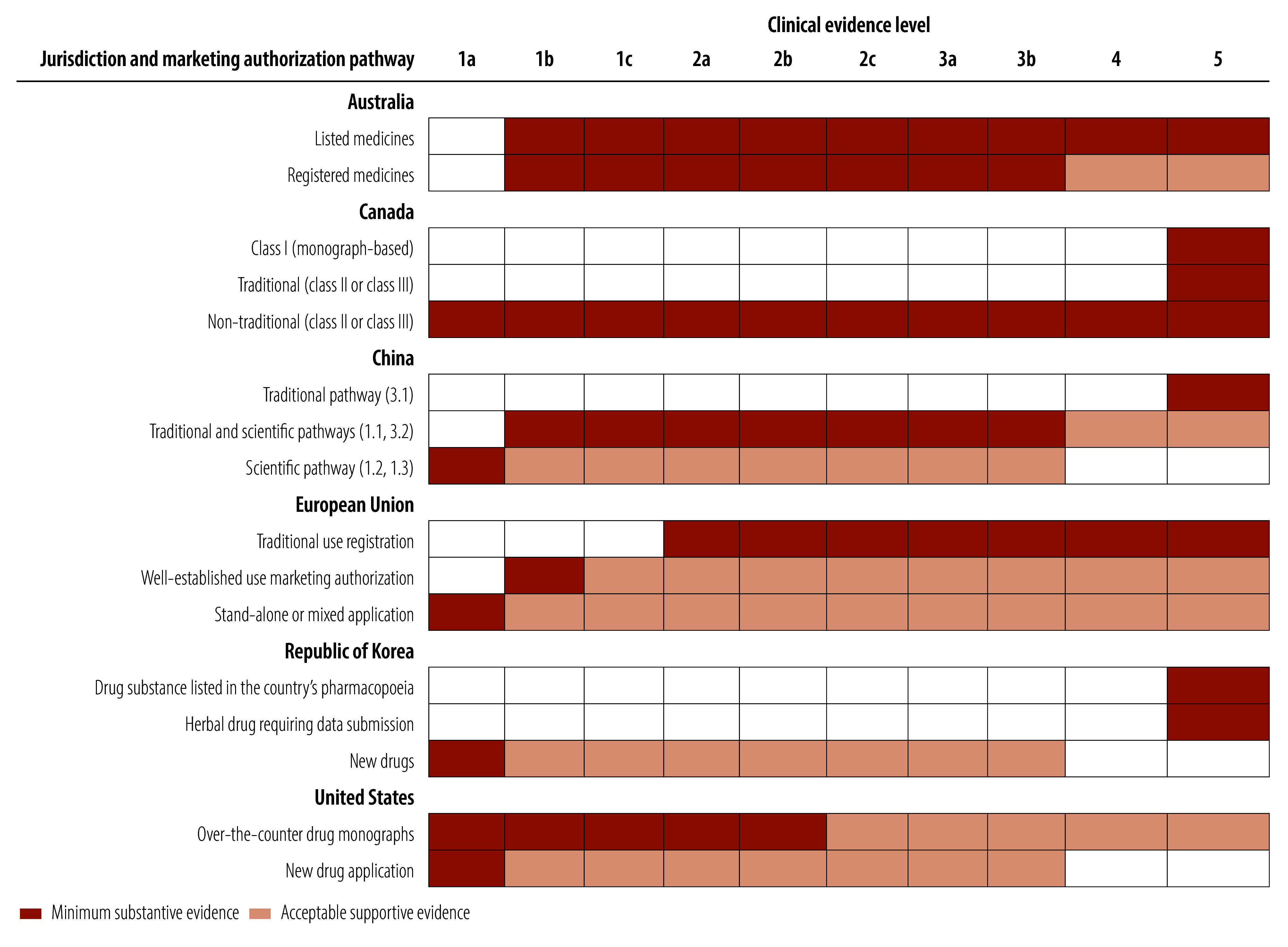
Clinical evidence levels required for traditional medicines to receive marketing authorization, by jurisdiction

Notes: data were retrieved from publicly available official sources (details provided in online repository).^14^ Substantive clinical evidence is evidence to demonstrate the efficacy and safety of a product for its intended use. Supportive evidence is supplementary information for regulatory decision-making but that is not sufficient to independently establish a product’s efficacy and safety. Evidence levels 1a to 1c (highest level of evidence) come from high-quality randomized controlled trials and systematic reviews of randomized controlled trials. Evidence levels 2a to 2c come from cohort studies, systematic reviews of cohort studies and outcome studies. Evidence levels 3a and 3b: come from case–control studies and systematic reviews of such studies. Evidence level 4 comes from case series and lower-quality cohort or case–control studies. Evidence level 5 (lowest level of evidence) comes from expert opinions, traditional use and fundamental principles.^12^^,^^13^

Appropriate evidence requirements for the marketing authorization of traditional medicines balance safety, efficacy and accessibility. Considerable variation exists between different jurisdictions in the level of substantive clinical evidence required and the regulatory flexibility based on the human-use history **(**Fig. 3 and online repository).^14^

From the perspective of the minimum substantive evidence required, pathways based on clinical study (registered medicines in Australia, scientific pathway in China, well-established use authorization or stand-alone or mixed application in the European Union, new drug pathway in the Republic of Korea, and over-the-counter drug monographs or new drug application in the United States) mandate the provision of the highest level of clinical evidence (level 1a–1b)^12^^,^^13^ for market approval, which refers to high-quality randomized controlled trials or systematic reviews of randomized controlled trials. Traditional knowledge-based pathways (listed medicines in Australia, all three pathways for natural health products in Canada, traditional pathway in China, traditional use registration in the European Union, and drug substances listed in the Pharmacopoeia of the Republic of Korea or herbal drug requiring data submission) accept level 4–5 evidence as substantive evidence. Notably, all regulatory frameworks recognize a lower level of supportive evidence to complement substantive evidence, considering the historical foundations of traditional medicines.

### Approvals of traditional medicine

The number of successful applications for marketing authorization also differed substantially across jurisdictions **(**Table 4 available at: https://www.who.int/publications/journals/bulletin/ ). In Australia, after the enforcement of the Therapeutic Goods Regulations in 1990, the number of listed traditional medicine products (low-risk products approved based on sponsor self-certification) has steadily increased, reaching 11 511 products by 2024, whereas the number of registered traditional medicine products (higher-risk products requiring full assessment through Therapeutic Goods Administration) remains relatively low (130 approved products). Similarly in Canada, the number of approvals has steadily increased since the implementation of Natural Health Products Regulations in 2004, with 130 989 Class I (monograph-based) products, 13 954 traditional products and 98 851 non-traditional products approved to date. However, Australia’s complementary medicines and Canada’s natural health products include a substantial proportion of non-herbal products: only 50.9% in Australia (737/1449) and 21.0% in Canada (3210/15 255) were classified as herbal products in 2023. Canada’s high approval volume of natural health products partially comes from its risk-based framework: over 70% of submissions on natural health products fell under Class I,^19^ which were approved within 60 days by using 315 pre-cleared monographs.^20^^–^^22^ The process was also facilitated by site licensing, electronic submission and a dedicated over-the-counter pathway with standardized but lower evidence requirements, while ensuring quality and safety.

**Table 4:** Number of traditional medicines receiving market authorization in Australia, Canada, China, Republic of Korea, United States of America and the European Union, by pathway and year

Pathway	Regulation	Year																																					
		1983	1985	1989	1990	1991	1992	1993	1994	1995	1996	1997	1998	1999	2000	2001	2002	2003	2004	2005	2006	2007	2008	2009	2010	2011	2012	2013	2014	2015	2016	2017	2018	2019	2020	2021	2022	2023	2024
**Australia**																																							
Listed medicines	1990: Therapeutic goods regulation came into effect				0	104	17	15	9	1	37	10	26	33	27	35	35	43	109	76	99	94	94	110	139	135	135	160	306	277	303	414	585	890	1 091	1418	1353	1443	1888
Registered medicines					0	51	0	1	2	0	0	3	0	3	0	1	1	0	1	0	1	0	2	1	2	1	4	2	3	1	4	5	4	5	10	6	4	6	6
**Canada**																																							
Class I (monograph-based)	2004: Natural health products regulations came into effect.																		116	1172	1338	2091	3593	3031	5255	5139	3969	5074	7746	9735	7285	7208	8692	7726	15 857	8948	8901	7554	10 559
Traditional (class II or III)																			32	20	38	294	554	818	635	652	2373	1503	1152	492	497	543	449	315	712	795	911	480	689
Non-traditional (class II or III)																			167	275	512	697	1525	2595	1970	3517	7974	6786	7290	6959	6978	5614	6377	4963	5 706	7945	7364	7221	6416
**China**																																							
Traditional pathway (3.1)	2020: Traditional Chinese medicine evaluation and approval system reformed, including registration categories																																		0	0	0	2	7
Traditional and scientific pathways (1.1, 3.2)																																			0	0	7	3	4
Scientific pathway (1.2, 1.3)																																			0	0	2	0	1
**European Union**																																							
European Union herbal monograph	2004: Herbal directive (2004/24/EC) introduced simplified registration procedure																		0	0	0	0	1	108	12	16	4	6	7	0	1	3	7	1	0	1	0	0	0
European Union list entry																			0	0	0	0	0	11	0	0	0	0	4	0	0	0	0	0	0	0	0	0	0
Public statement																			0	0	0	0	0	7	3	2	1	0	5	2	0	1	1	1	0	0	0	0	0
**Republic of Korea^a^**																																							
Listing	2005: Pharmaceutical affairs act revised																			3789	4241	4126	2214	2002	2069	6622	3235	206	231	2005	1031	43	457	1472	337	269	179	186	45
Approval																				35	37	44	27	33	14	89	22	14	29	67	31	476	42	59	64	21	8	12	2
**United States**																																							
Over-the-counter drug monographs	1972: Over-the-counter drug review established^b^	2	1	1	1	1	1	1	0	2	2	2	2	0	0	0	0	0	0	0	0	0	0	0	0	0	0	0	0	0	0	0	0	0	0	0	0	0	0
New drug application	2006: Botanical drug development guidance issued																				1	0	0	0	0	0	1	0	0	0	0	0	0	0	0	0	1	1	0

EU: European Union.

^a^ Listing includes products listed in the Pharmacopoeia of the Republic of Korea or requiring data submission, while approval refers to new drugs granted full marketing authorization.

^b^ There were no approvals between 1972 and 1983.

Note: data were retrieved from publicly available official sources (details provided in online data repository).^14^

In China, after the regulatory reform of traditional Chinese medicines in 2020,^23^ nine, 14, and three traditional medicine products have been approved under the traditional pathway, traditional and scientific pathways and scientific pathway, respectively. Since the introduction of a simplified registration procedure for traditional herbal medicines in 2004, the European Union has issued 12 herbal monographs, two list entries and nine public statements supported by the established standards of the European Medicines Agency. In the Republic of Korea, after the 2005 revision of the Pharmaceutical Affairs Act, annual market entries fluctuated, with listing products (including pharmacopoeia and data submission) peaking in 2011 (34 759 listed products currently) and approved new drugs peaking in 2017 (1126 approved products currently). In the United States, over-the-counter drug review began in 1972, and 16 herbal substances have been included in the over-the-counter monograph system (aloe vera, coal tar, camphor, cocoa butter, colloidal oatmeal, corn oil emulsion, echinacea, ephedrine, glycyrrhiza, hydrogenated soybean oil, menthol, panax ginseng, pyrethrum, rhubarb, St John’s wort and witch hazel). After the publication of Botanical Drug Development Guidance in 2006 in the United States, three botanical medicines were approved through the new drug application pathway (Filsuvz®, Fulyzaq® delayed-release tablets and Veregen® ointment); and one (NexoBrid®) was approved through the biologics licence application pathway. Overall, the number of successful applications under traditional knowledge-based pathways tends to be higher than through pathways based on clinical studies.

### Successful multiple registration

Compound danshen dripping pills is a proprietary multiherb product composed of *Salvia miltiorrhiza*, *Panax notoginseng* and synthetic borneol. These herbs have long been used in traditional Chinese medicine to promote circulation and relieve chest discomfort caused by blood stasis. Compound danshen dripping pills were first approved in China in 1995 under the category of new compound formulations, and are now listed as a reimbursable List A prescription drug (Table 5).^24^^,^^25^ In Canada, this product was approved in 2024 as a natural health product through the traditional pathway (Class II or Class III) but is not reimbursed through the public health system. In the Republic of Korea, the product was approved in 2002 as a non-prescription herbal product requiring data submission; it is also not reimbursed.

**Table 5:** Regulatory approval and market access pathways of compound danshen dripping pills, Australia, Canada, China, Republic of Korea, United States of America and the European Union

Jurisdictions^a^	Regulatory status	Product number	Date of licensing	Marketing authorization pathway	Product category	Reimbursed by national insurance
Australia	GMP accreditation obtained	NA	NA	Eligible for therapeutic goods administration GMP via EU–Australia mutual recognition agreement (based on EU GMP certificates)	NA	No
Canada	Approved	80 135 681	4 Nov 2024	Traditional (Class II or III)	Self-care product	No
China	Approved	Z10950111	1 Jan 1995	Former class III traditional Chinese medicine compound preparation (now equivalent to Class 1.1 innovative drug)	Prescription drug	Yes (list A)
European Union	GMP accreditation obtained	NA	NA	Multiple EU GMP certifications^b^	NA	No
Republic of Korea	Approved	200 209 620	4 Jan 2002	Herbal drug requiring data submission	Non-prescription drug	No
United States	Under development	Investigational new drug application: Dantonic® (T89)	NA	Investigational new drug approved in 1998; phase III trial completed in 2016; new drug application pending	Investigational new drug	No

EU: European Union; GMP: good manufacturing practice; NA: not applicable.

^a^ Note: We retrieved information from publicly accessible regulatory databases, including Health Canada, National Medical Products Administration (China), EudraGMDP (EU), Ministry of Food and Drug Safety (Republic of Korea) and United States Food and Drug Administration, as well as official disclosures from manufacturers.

^b^ United Kingdom of Great Britain and Northern Ireland in 2011, Denmark in 2012 and Kingdom of the Netherlands in 2017.

While compound danshen dripping pills have not yet received marketing authorization in the United States, investigational new drug status was granted in 1998 (Dantonic®, code T89) and the phase III trial (NCT01659580) was completed in 2016; approval by the Food and Drug Administration (FDA) is pending. In the European Union, the manufacturer obtained multiple good manufacturing practice certifications in Denmark (2012) and Kingdom of the Netherlands (2017), which facilitated regulatory access. Australia recognizes these certifications under mutual recognition agreements, although compound danshen dripping pills have not yet been marketed there.

## Discussion

### Quality, safety and efficacy

In 2023, the global market size of traditional medicines was 144.68 billion United States dollars (US$) and is projected to reach US$ 694.22 billion by 2030, with a compound annual growth rate of 25.3%.^26^ This growth is driven by the increasing public awareness of natural therapies, prevalence of chronic diseases and emphasis on preventive health care globally.^27^^,^^28^

Jurisdictions have developed three main categories of regulatory frameworks. The first type, used by Australia and Canada, regulates traditional medicines as a special category, covering a broader range of substances with lower risk. These products are typically sold as non-prescription products, and the simpler requirements resulted in higher numbers of approvals than in jurisdictions requiring full drug evaluation for traditional medicine products. Australia adopts a pragmatic, risk-based assessment approach that enables high volume of products meeting appropriate safety standards to be approved each year, thereby improving patient access.^29^ Canada seeks a balance between the recognition of traditional practices and risk management.^30^

The second type of regulatory framework, adopted by the European Union and the United States, applies a biomedicine regulatory framework with specific adjustments for traditional products. The European Union, the largest consumer of commercially produced herbal medicinal products,^31^ offers a simplified registration pathway for herbal products with longstanding use, limited to non-prescription products and excluding injectables. In the United States, data on traditional use may support investigational new drug applications for phase I and II trials, but full non-clinical and clinical data are required for approval of a new drug application.^32^ Although the FDA allows botanical substances listed in over-the-counter monographs, it requires published evidence of general recognition, including adequate and well-controlled clinical trials. These trials are challenging for complex multicomponent products that rely solely on traditional use.

The third type of regulatory framework, used by China and the Republic of Korea, adopts a more region-specific framework based on local practices. China offers three pathways (traditional, traditional and scientific, and scientific) to ensure a comprehensive use of evidence for evaluation,^33^ allowing traditional medicine products initially to be sold only as prescription medicines. In the Republic of Korea, herbal products listed in specific herbal medicine books can follow a simplified registration pathway, and products can be registered as either prescription or over-the-counter medicines depending on safety and potential risks.

As the market expands, regulatory authorities face growing challenges to ensure product safety and efficacy. Key priorities should include: preventing contamination and adulteration (e.g. heavy metals, pesticides or undeclared pharmaceuticals) through enhanced testing standards of raw materials and finished products; ensuring batch-to-batch consistency through the implementation of good manufacturing practice and standardized extraction processes; managing herb–drug interactions by establishing pharmacovigilance mechanisms; and curbing misleading health claims through rigorous review of marketing materials and labelling.

### Strengthening the evidence

A major barrier to the integration of traditional medicine products into mainstream health care is the lack of high-quality evidence.^4^^,^^34^ Many such products have been used for a long time and are believed to have lower risks.^35^^,^^36^ However, this reliance on anecdotal data often results in inconsistent clinical outcomes.^37^ In the advocacy of evidence-based policy-making,^38^^,^^39^ clinical trials of traditional medicine products face challenges in deciding specific patient populations, interventions, comparators and outcome measures to draw clear scientific conclusions that are communicable to general medical readers.^40^

High-quality evidence is the cornerstone for the development of traditional medicine products.^36^^,^^41^ To strengthen the evidence base, several key strategies can be considered. First, refining traditional indications in a well-defined target population (e.g. based on age, symptom patterns or disease stage), together with establishing clear diagnostic criteria and standardizing intervention protocols, can enhance the reproducibility and comparability of research outcomes.^40^ Developing stricter quality-control methods is important to ensure batch-to-batch consistency of traditional medicines.^42^^–^^44^ Adopting patient-centred outcome measures,^45^ such as validated clinical outcome assessments, can provide accurate and meaningful evaluations of the efficacy of traditional medicines in clinical studies. Second, careful consideration of robust real-world evidence can help bridge the gap between traditional practice and scientific research.^46^ Real-world studies, including pragmatic clinical trials and well-designed prospective observational studies, can better capture the complexity of traditional medicine practice and generate clinically relevant data,^47^^,^^48^ which could be used to replace preclinical studies. For instance, China has implemented guidelines for real-world studies of traditional Chinese medicines, emphasizing the use of human-experience data to support clinical evaluation.^49^

### Regulatory gaps and policy implications

Our analysis highlights issues that hinder the global development of traditional medicine products and the lack of regulatory reciprocity which limits equitable access to these medicines. Although all six jurisdictions we assessed have established comprehensive independent regulatory pathways, the difference in evidence requirements, classification systems and lack of mutual recognition creates barriers to harmonization (Table 6).

**Table 6:** Acceptable evidence, gaps and policy implications for traditional medicine products, Australia, Canada, China, Republic of Korea, United States of America and the European Union

Country or region	Traditional evidence accepted	Mutual recognition mechanism between jurisdictions	Identified gaps	Future policy implications
Australia	Yes (listed pathway)	Yes (good manufacturing practice via mutual recognition agreements)	Traditional evidence supporting only listed medicines, which have limited evaluation, thus restricting international recognition.	Establish a rigorously assessed category for traditional medicines with clear evidence standards to enhance international credibility and support global mutual recognition
Canada	Yes (for traditional claims)	No (limited pharmacopoeia recognition)	No defined pathway for prescription-grade traditional medicines with higher evidence standards	Strengthen evidence standards and create a regulatory pathway for high-standard traditional medicines; support international convergence on evaluation criteria
China	Yes (3.1); partly (1.1, 3.2)	No	Lack of harmonized evidence standards; limited international acceptance	Promote alignment with international standards for evidence qualification and facilitate global acceptance of Chinese traditional medicines through bilateral or multilateral mechanisms
European Union	Yes (for traditional use and well-established use)	Yes (within EU)	Fragmented national implementation; limited interface with global systems	Harmonize national interpretations of EU frameworks; establish bridges to global regulatory systems to facilitate international recognition of products approved via traditional use and well-established use pathways
Republic of Korea	Yes (for pharmacopoeia and data-submission herb)	No	Limited transparency in traditional use assessment; unclear classification boundaries	Enhance clarity in pathway criteria; promote cross-border alignment with international standards; increase transparency in evaluation and classification systems
United States	Limited (full clinical evidence required)	No	Stringent scientific standards limiting the integration of traditional evidence	Facilitate integration of qualified traditional evidence into regulatory review processes; participate in global dialogues on balanced evidence frameworks

Note: data were retrieved from publicly available official sources.

Standardized traditional medicine categories should be established for rigorous evaluations of these products. Evaluations should consider a wider range of credible evidence through mutual recognition of pharmacopoeias and establishing regional recognition agreements. A globally coordinated, tiered, risk-based international framework that integrates traditional and scientific evidence is needed to facilitate the safe and effective integration of traditional medicines into health-care systems.
